# Preliminary Outbreak Investigation and Spatial Epidemiology of Rabbit Hemorrhagic Disease Outbreak in Nigeria

**DOI:** 10.3389/fvets.2021.771599

**Published:** 2022-01-05

**Authors:** Muftau Oyewo, Ahmad I. Al-Mustapha, Bukola A. Richards, Lateefah Abdulkareem, Taiwo Olasoju, Sufiyan M. Babale, Hamza Khalid, Clement Meseko, Muhammad S. Balogun

**Affiliations:** ^1^Department of Veterinary Services, Kwara State Ministry of Agriculture and Rural Development, Ilorin, Nigeria; ^2^Nigerian Field Epidemiology and Laboratory Training Program, Abuja, Nigeria; ^3^Department of Veterinary Public Health and Preventive Medicine, Faculty of Veterinary Medicine, University of Ibadan, Ibadan, Nigeria; ^4^UMR 1289, Faculty of Pharmaceutical Sciences, Universite de Tours, Tours, France; ^5^Faculty of Veterinary Medicine, University of Ilorin, Ilorin, Nigeria; ^6^Department of Veterinary and Pest Control Services, Federal Ministry of Agriculture and Rural Development, Abuja, Nigeria; ^7^Department of Community Medicine, Ahmadu Bello University, Zaria, Nigeria; ^8^Viral Research Laboratory, Veterinary Research Institute, Lahore, Pakistan; ^9^Regional Laboratory for Animal Influenza and Transboundary Animal Diseases, National Veterinary Research Institute, Vom, Nigeria; ^10^African Field Epidemiology Network, Abuja, Nigeria

**Keywords:** RHD, Kwara RHDV-2, outbreak investigation, spatial epidemiology, rabbits

## Abstract

The outbreak of highly contagious transboundary rabbit hemorrhagic disease (RHD) in Nigeria has a severe socio-economic impact on the rabbit industry. We present the outbreak investigation and spatial epidemiology of the first confirmed RHD outbreak in Nigeria from a field survey of 28 stochastic outbreaks in Kwara State, north-central Nigeria. A total of 1,639 rabbits died from 2,053 susceptible rabbits. The serotype “RHDV-2” was detected in tissue samples from some of the outbreaks. The case fatality rate of the RHDV-2 outbreak was 79.8%. The source of the outbreak is still unknown. Most (71.4%) of the farmers had introduced new rabbits into their farms 1–2 weeks before the outbreak. Most of the farmers practiced biosecurity measures such as farm fencing (83.1%) and routine disinfection of the farm materials (53.6%). However, only 17.8% of the farmers enforced movement restrictions into their farms. Some of the farmers (42.8%) had restocked their farms after being affected by the RHD outbreak and 75% of all those farmers that have restocked had used the RHD vaccine. There was no statistically significant association between adherence to biosecurity measures and the RHD outbreak in affected farms (*p* = 0.408). However, the introduction of new rabbits into rabbit farms significantly pre-disposed farms to the RHD outbreak (*p* < 0.001). There is a need for active surveillance of RHD across the country to ensure efficient and effective tracking, monitoring, and control of the disease. Equally, understanding the genetic diversity of the Lagoviruses in Nigeria that cause RHD to aid vaccine development is of utmost importance to prevent future RHD outbreaks.

## Introduction

Rabbit hemorrhagic disease (RHD) is a devastating non-zoonotic disease of rabbits caused by the rabbit hemorrhagic disease virus (RHDV), a Lagovirus of the Caliciviridae family, which is a positive-sense single-stranded RNA virus. The first clinical report of rabbit hemorrhagic disease was in the 1980s in China where the disease killed 14 million European Angora rabbits in 9 months (1). In less than a year, RHD killed 140 million domestic rabbits in China and spread over an area of 50,000 km^2^ (2). Confirmed rabbit hemorrhagic disease virus-2 [RHDV-2 (a variant strain)] outbreak has been reported in domestic rabbits from Benin Republic (3), Egypt (4), Saudi Arabia (5), United Kingdom (6), Portugal (7), and several other countries. However, before 2020, there have been no confirmed cases in Nigeria making it difficult to trace the source of the outbreak, the spatial and temporal spread as well as the socio-economic impact on rabbit farmers. The disease is transmitted by direct contact with infected animals, body fluid, carcasses, fomites, or vectors like flies, fleas, and mosquitoes (8, 9).

The RHDV-2 has 100% morbidity and 60–90% mortality rates (10). Currently, three strains of RHDV have been identified: RHDV, RHDVa, and RHDV-2. Similarly, three clinical forms of the RHD have been identified: the per-acute, acute and sub-acute forms with the incubation period ranging from 3 to 5 days (11). In the per-acute form, infected animals show no signs and die suddenly (10, 12). In acute infections, the rabbits are observed to be inactive, reluctant to move, with bloody discharge from all orifices as well as blood in feces or urine and may develop a fever with temperature up to 42°C (10). Rabbits with the acute form die within 12–36 h from the onset of fever (12). The sub-acute forms present in the same way but with milder symptoms and most rabbits survive. The clinical signs include lethargy, anorexia, weight loss, and jaundice. Death occurs within 1–2 weeks after the onset of symptoms due to liver failure (10). Rabbits infected with RHDV2 are more likely to show sub-acute or chronic signs than those infected with RHDVa (13). Postmortem signs seen in rabbits with RHD are extensive hepatic necrosis, jaundice, multifocal hemorrhages, splenomegaly, bronchopneumonia, pulmonary hemorrhage or edema, and myocardial necrosis (10, 14).

The first laboratory-confirmed RHDV outbreak that was reported to the Office International des Epizooties (OIE) from Nigeria was recorded on the 13th of October 2020 (15). The source of the outbreak is still unknown. The index case of the virus was in Kwara State, Nigeria, where over 300 rabbits were affected on the farm. Subsequently, confirmed outbreaks have been reported in Oyo and Ogun states (15). A national response was instituted by the federal government to stop the further spread of the disease and to enlighten farmers on biosecurity measures which they should adopt on their farms. As of November 30th, 2020, a total of 1,639 self-reported rabbit deaths have been recorded from 2,053 susceptible or infected cases in Nigeria (16). Hence, this study conducted an outbreak investigation and assessed the Spatio-temporal distribution of the RHD among 28 infected farms in Kwara State. Furthermore, the study identified the risk factors and assessed the economic impact of the outbreak in Kwara State.

## Materials and Methods

### Study Settings

**Figure 1:**
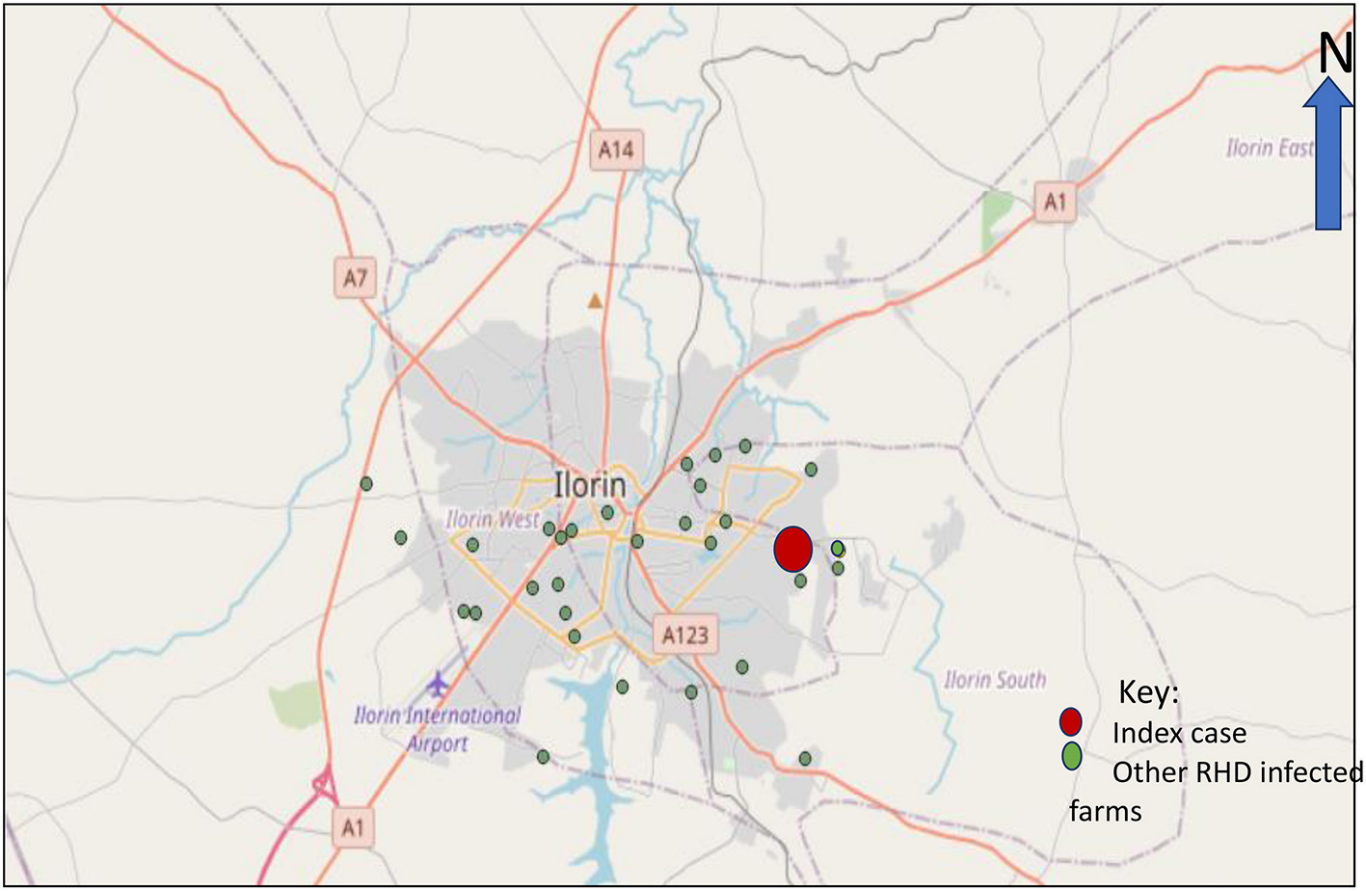
Map of Ilorin showing the index case and geographical spread of the reported outbreak.

Kwara State is one of the states in the Northcentral zone of Nigeria. Crop farming is the major occupation of its people. Of recent, the livestock sector has boomed in the state, and the state has at least 60 known rabbit farms. This study was conducted in 28 RHD-infected farms in the Ilorin metropolis (made up of 4 local government areas), between October and November 2020 based on the pre-existing definitions of a suspected, probable, or confirmed case. The infected farms (Figure 1) were clustered across 4 Local Government Areas (LGAs) of the Kwara Central senatorial zone.

### Case Definition and Case Finding

A case of RHD was defined as multiple cases of sudden death in rabbits after a short period of fever, lethargy, anorexia, characteristic hepatic necrosis, and multi-organ hemorrhages at necropsy (10). Other non-specific signs include nervous and respiratory signs and disseminated intravascular coagulopathy in the trachea, lungs, and liver.

A confirmed case is any suspected case that has been confirmed in the laboratory. A probable case is one that was not confirmed in the laboratory but has direct epi-linkages with a confirmed case. A suspected case is one in which some of the signs of RHD have been reported but no epi-linkages to a probable or confirmed case have been established. In addition, we conducted a retrospective case search based on the clinical history, case definition, and necropsy findings.

### Descriptive Epidemiology

Based on the case definition, the index case was traced back to the necropsy finding of a rabbit that died on the 22nd of June 2020 (Figure 1). The tentative diagnosis was made at the State Veterinary Clinic after the correlation of clinical cases, postmortem findings coupled with high morbidity and mortality rates among rabbits. Blood and tissue samples (liver, kidney, and mesenteric lymph nodes) were collected from 16 rabbits obtained from 4 infected local government areas at postmortem, and laboratory tests confirmed RHDV-2. All other RHD infected farms within these four affected LGAs were linked *via* direct epi-linkages such as direct animal transactions with an infected farm, direct contact with other RHD infected farm owners, and in certain cases through fomites such as commercial feed bags when an infected and a susceptible farm shared rabbit feed. In addition, most of the affected farms in each LGA were located within a 5 km radius and were linked *via* major roads and exchange rabbits for mating purposes and sales.

### Data Collection Tool

A structured questionnaire was administered in one-on-one interviews with 28 affected rabbit farmers to identify the source of the virus, the spread within the state, possible modes of transmission of the disease within the state, and the economic impact to the farmers. The questionnaire was divided into three segments. Section A focused on the rabbit farmer socio-demographic characteristics whereas sections B and C obtained information on rabbit husbandary practices and the epidemiology of the rabbit hemorrhagic disease, respectively. The questionnaire was pre-tested on 6 rabbit farmers in Ilorin and screened to certify its content and face validity. The reliability of the survey instrument was assessed using the Cronbach's Alpha test (with a score of 0.79).

### Data Collection and Analysis

Field data were collected from RHD infected farms between October 19th and November 11th, 2020. The data were collected by veterinarians (from the Ministry of Agriculture and Rural Development, Ilorin-Nigeria) while conducting an outbreak investigation of the 2020 RHD outbreak. The data were summarized using descriptive statistics like frequencies and proportions for qualitative data and quantitative data were expressed as mean. We used Minitab v.17 to compute the descriptive statistics as well as test of association between variables (Chi square). Finally, we used QGIS v3.10.2 to compute the spatial spread of the RHD outbreak in Kwara State.

## Results

### Clinical Presentation and Gross Findings

The most frequently reported clinical signs by affected farmers were reduced appetite (100%), sudden death with no obvious symptoms (71.4%), mucoid-tinged feces (50%), lacrimal discharge (7.1%), lethargy (75.0%), violent shaking of the rabbits few minutes before death (25.0%), and bleeding *via* the nasal and oral orifices (42.9%). Postmortem examination was conducted on a total of 79 rabbits (from 14 commercial farms and 9 backyard rabbit farms) at the state veterinary clinic, Ilorin-Nigeria. Most of the carcasses (72.1%) presented for necropsy were exotic breeds (because they are more expensive, and their owners wanted to prevent further mortality) and weighed 4 kg and above. There were severe multifocal petechial hemorrhages of the trachea (51.8%), lungs (77.2%), and liver (81.0%). Other lesions seen at necropsy were frothy exudate in the trachea (34.1%), fused pericardiac sac (8.8%), and localized lymphadenitis (hepatic and mesenteric−13.9%).

A total of 1,639 rabbits died of RHD in 16 reported outbreaks geographically spread across 28 farms within the state. The case fatality rate of the RHDV-2 outbreak in Kwara State was 79.8%.

### Laboratory Confirmation of RHDV

The samples were positive for RHDV-2 by quantitative polymerase chain reaction (RT-qPCR) targeting the VP60 gene.

### Spatial Epidemiology of the RHD Outbreak

**Figure 2:**
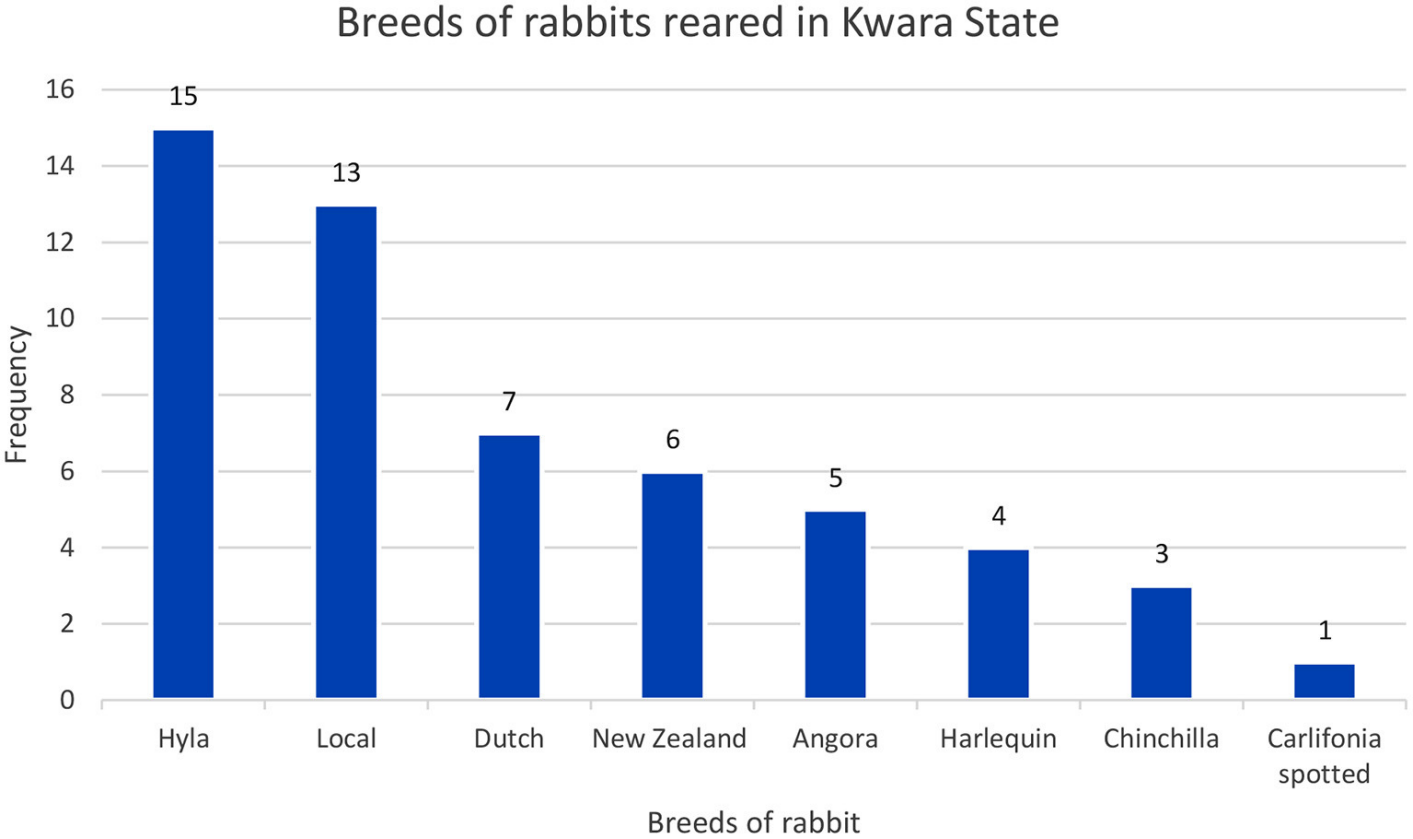
The breeds of rabbits commonly reared on farms visited (*n* = 28).

Most of the rabbit farmers were aged between 30 and 49 years (57.2%). Most affected farmers had tertiary education (67.9%) and had received formal training on rabbit production (71.4%) (Table 1). Half of the affected commercial farms had over 100 rabbits each that were mostly exotic breeds. Most rabbit farmers in the study area had both the exotic and local breeds of rabbits. More than half (53.5%) of the farmers reared the Hyla breed (Figure 2).

**Table 1:** Sociodemographic characteristics of Rabbit Farmers in Kwara State (*n* = 28).

**Variables**	**Frequency (%)**
**Age**	
20–29	4 (14.3)
30–39	8 (28.6)
40–49	8 (28.6)
50–59	6 (21.4)
>60	2 (7.1)
**Level of education**	
Secondary	9 (32.1)
Tertiary	19 (67.9)
**Formal training in Rabbitry**	
Yes	20 (71.4)
No	8 (28.6)

A total of 28 rabbit farms have been affected since the detection of the RHD outbreak in the index farm (Ilorin South LGA). The infected farms were in close proximity, had contact with index farm, bought rabbits from the same source with index farm, or traded with the index case.

Most rabbit farmers (53.6%) also reared poultry and all farmers reared at least one other animal. Most of the farmers had used antibiotics (67.9%), and some had used antiparasitic drugs (35.7%) in the last 30 days before the RHD outbreak in their respective farms. Most of the rabbits (92.3%) were fed with commercially sold feeds. Rabbit farmers had previously recorded coccidiosis (60.7%) and mange (50.0%) as the most commonly diagnosed diseases in their rabbits (Table 2).

**Table 2:** Characteristics of rabbit farms visited in Kwara state (*n* = 28).

**Variables**	**Frequency (%)**
**Population of rabbits**	
<20	11 (39.2)
21–50	2 (7.1)
51–70	0 (0.0)
71–100	1 (3.6)
>100	14 (50.0)
**Types of feed used**	
Commercial feed	26 (92.3)
Kitchen waste	2 (7.1)
Locally compounded feed	2 (7.1)
Vegetables alone	3 (10.7)
**Other animals reared on the farm**	
Poultry	15 (53.6)
Sheep	6 (14.3)
Goats	8 (25.0)
^*^Others	2 (7.1)
**Previously experienced diseases on the farm**	
Coccidiosis or other diarrheal diseases	17 (60.7)
Pneumonia or respiratory tract infections	11 (39.3)
Skin malformations	14 (50.0)
Intestinal worms	9 (32.1)
**Drugs used in the last month**	
Antibiotics	19 (67.9)
Anti-helminthics	11 (39.3)
Anti-parasitic (such as coccidiostat)	10 (35.7)
Multivitamins	6 (21.4)

* *One dog and one pig*.

Of the 28 affected rabbit farms included in this study, 71.2% of them had recently introduced new rabbits into their farms. Most of the newly introduced rabbits were either mostly exotic bucks (for mating) or does (for production). Only 3 of the RHD infected farms had introduced local rabbit breeds together with the exotic breeds into their farms before the outbreak (<2 weeks).

**Figure 3:**
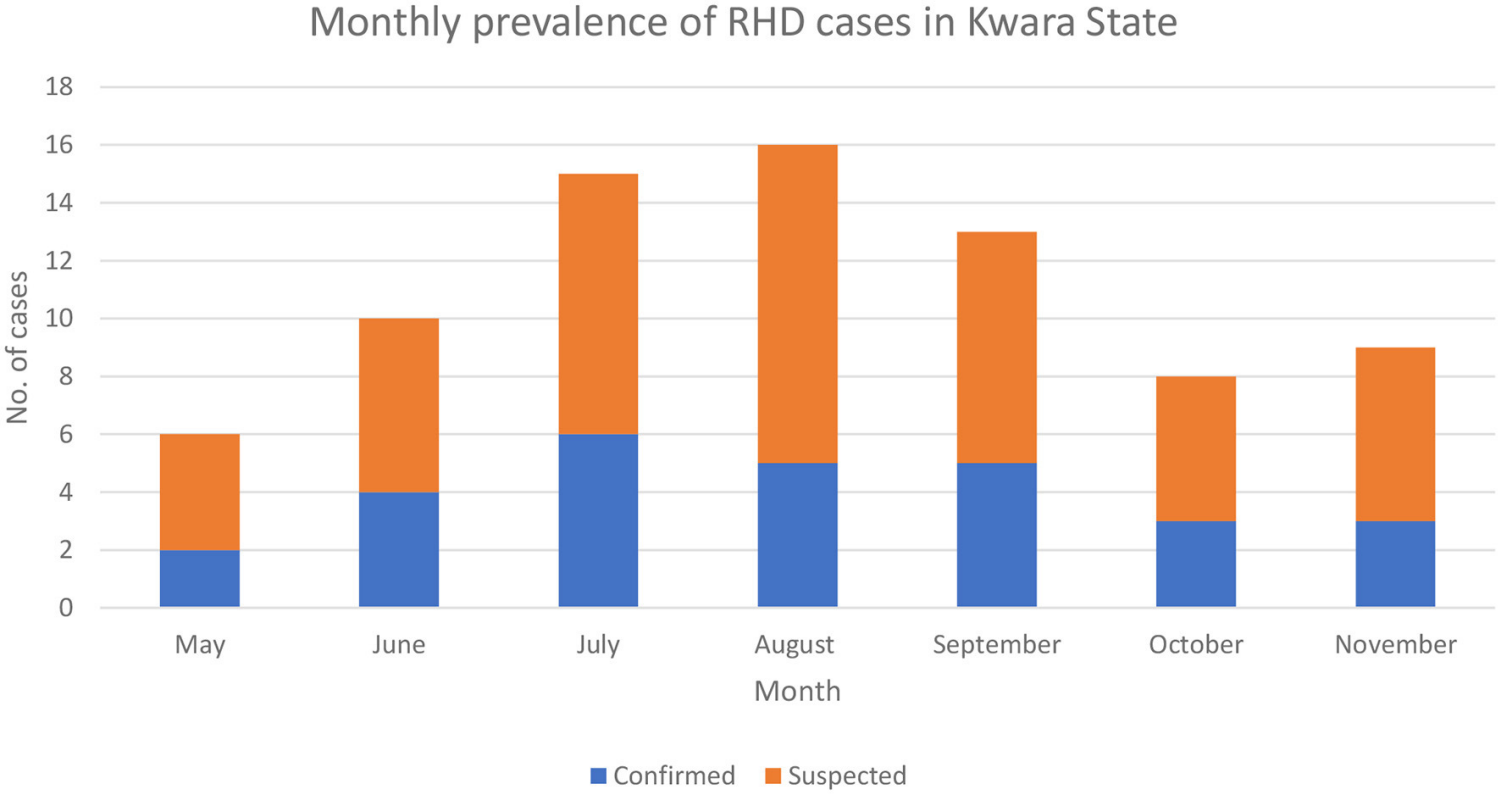
Monthly prevalence of suspected and confirmed RHD outbreaks per month in Kwara State as of 31st November 2020.

Although the laboratory confirmation of RHDV-2 was in September 2020, a review of case files based on clinical presentation and postmortem findings revealed that the index case of the RHDV outbreak in Kwara State was in June where the farmer lost all 32 rabbits on the farm. The highest number of reported outbreaks was in July (Figure 3). Older rabbits (>3 months) were mostly affected (71.2%). Three-quarters of the farmers culled their rabbits after the outbreak. Only 25.0% of the affected farmers reported the outbreak to their veterinarian.

Most of the farmers practiced biosecurity measures such as fencing of the farm area (83.1%, *n* = 23) and routine disinfection of the farm materials (53.6%, *n* = 15). However, only 2 (7.1%) farmers had foot dips at the entrance of their farms and only 17.8% of the farmers enforced movement restrictions into their farms. Some of the farmers (42.8%, *n* = 12) had restocked their farms after being affected by the RHD outbreak and 75.0% (*n* = 12) of all those farmers that have restocked had used the RHD vaccine. The use of biosecurity (67.9%, *n* = 18) and regular disinfection of the hutch (39.3%, *n* = 11) by farmers were the main preventive measures taken against future outbreaks of RHD on their farms (Table 3).

**Table 3:** Epidemiology of RHDV in Kwara state (*n* = 28 farms).

**Variables**	**Frequency (%)**
**Introduction of new stock to the farm**	
Yes	20 (71.4)
No	8 (28.6)
**Observed clinical signs^a^**	
Bloody or brownish diarrhea	5 (17.9)
Coughng	5 (17.9)
Sudden death (No notable sign)	20 (71.4)
Violent shaking of the rabbits	7 (25.0)
**Age of affected rabbits**	
0–6 weeks	2 (7.2)
6–12 weeks	6 (21.6)
3 months and above	20 (71.2)
**Case fatality rate**	
0–20%	4 (14.2)
21–40%	1 (3.6)
41–60%	3 (10.8)
61–80%	11 (39.2)
80–100%	9 (32.1)
**Actions after outbreak**	
Fumigating the hutch	8 (28.6)
Reported to the veterinarian	7 (25.0)
Culled the rabbits	21 (75.0)
Selling the rabbits alive	4 (14.3)
**Steps taken to prevent further outbreaks**	
Perimeter fencing	19 (67.9)
Disinfection of hutch	11 (39.3)
Praying	2 (7.1)
Regular hand washing	7 (25.0)
**General disease prevention measures observed**	
Placement of foot dips at the farm gate and hutch entrance	2 (7.1)
Routine disinfection of farm facilities	15 (53.6)
Fencing of farm	23 (83.1)
Restricting movement of visitors	5 (17.8)
**Presence of stagnant water on the farm**	
Yes	16 (57.1)
No	12 (42.8)
**Financial implications of the outbreak (Naira)**	
<50,000	4 (14.2)
51,000–250,000	6 (21.4)
251,000–500,000	3 (10.7)
501,000–750,000	14 (50.0)
>1 million	4 (14.2)
**Restock farm after the outbreak**	
Yes	12 (42.8)
No	16 (57.2)
**RHD vaccine use (*n* = 12)**	
Yes	9 (75.0)
No	3 (25.0)

a *Multiple responses*.

There was no statistically significant association between adherence to biosecurity measures and the RHD outbreak in affected farms (*p* = 0.408). Similarly, farmers that restricted movements into their rabbit farms also had outbreaks of RHD. However, the introduction of new rabbits into rabbit farms significantly pre-disposed farms to the RHD outbreak (*p* < 0.001).

The basic farm biosecurity measures especially the rapid disinfection of hutches and the movement restriction impacted the case fatality rate of rabbits within farms but not the occurrence of the RHD in their farms (*p* < 0.001).

## Discussion

The outbreak of transboundary animal diseases (TADs) such as RHD has a serious socio-economic impact on the livelihood of small-scale farmers. Although several un-investigated reports have described the occurrence of an acute hemorrhagic disease of rabbit that was imported from Benin Republic into Nigeria (17), it was never investigated, diagnosed, monitored, and reported to the OIE. This study shows that RHD is a devastating disease that spreads very fast from farm to farm, possibly as a result of mechanical transmission through the common practice of animal sharing for mating purposes as well as other fomites.

The disease has a very high morbidity (100%) and mortality (79.8%) rates occurring in clusters within affected areas and the effect tends to be less in farms that have considerable distance from other farms signifying the role of fomites in its transmission. Similar to outbreaks in Ghana (18), we observed a seasonal peak of RHD infections in July, which may be attributed to the mating season in wild hares which brings into close contact hares from different areas as observed in the outbreak in Portugal. Similar results were observed in which some rabbit farmers introduced bucks to other farms for mating. The poor veterinary expert-seeking behavior of the farmers could be responsible for the increased incidence of the disease after the initial outbreak as most farmers resulted to self-medication using antibiotics; this may portend a danger of antimicrobial residues in humans who consume such animals.

Kwara state has an international border with Benin Republic. Hence, TADs such as RHD could spread easily into susceptible rabbit populations in Nigeria. Several factors favor the introduction of TADs into Nigeria. These factors include the poor animal disease surveillance system and the lack of animal movement control across the land borders.

Our findings showed that several factors were common among RHD-infected farms. These include: 1. The introduction of a new rabbit(s) into farms was the single most important risk factor for the introduction of the RHDV-2 into susceptible rabbits. 2. There was no difference in sex distribution of affected rabbits as morbidity and mortality cut across both male and female rabbits. 3. Some of these farms had stagnant water, hence the possibility of transmission *via* vectors such as insects and houseflies. 4. Farmers within clusters (i.e., close proximity) had unrestricted meetings, hence could serve as mechanical fomites. 5. Younger rabbits <2 months old were less affected than older rabbits. In affected farms, rabbits were culled, the environment fumigated, and reported to the veterinarian only in a few cases.

The most common clinical presentation seen among these rabbits and the CFR were similar to reports of OIE (14), and Happi et al. (19).

Although rabbits were kept in cages, domestic and migratory birds could have access to rabbit cages which could serve as sources of infection for rabbits. The training that most of the farmers reported to have received in rabbit production, coupled with their high level of education could be responsible for some of the biosecurity practices noticed in most farms visited. The use of antibiotics by the farmers is similar to an earlier study of Chah et al. (20) who reported high unprescribed use of antibiotics in rabbits in Enugu State. Similarly, Chah et al. (20) also reported that rabbits were mostly affected by sniffles, mange, and diarrhea. The un-prescribed use of antibiotics could result in the emergence of drug-resistant micro-organisms, some of which could pose a public health threat (21).

Although, the official government policy is “no vaccination against RHD,” some of the rabbit farmers have restocked their farms and vaccinated their rabbits using foreign vaccines. This is highly discouraged as the efficacy of such foreign vaccines on local rabbit populations has not been ascertained and might lead to the introduction of novel strains into Nigeria. In addition, the vaccine only confers immunity for 12 months (22) to 14months (23) and it is believed that vaccination could make RHD endemic (24, 25). Hence, several countries including Nigeria discourage the use of vaccines until a comprehensive epidemiology is carried out to determine causative serotypes in order to adequately provide guidance.

Moreover, it may be more helpful to develop in-country vaccines based on circulating strains. Our findings Figure 1 showed the increasing spread of the virus over time in the state from the index case. Hence, it is essential to carry out statewide active surveillance of RHD to know the true prevalence and incidence of the disease. The RHDV-2 outbreak has devastated farmers with economic losses running into millions of Naira. Hence, the affected farmers should be adequately compensated by the government.

The main strength of the study was its novelty as the first confirmed RHD outbreak in Nigeria and the major limitation was the small sample size.

## Conclusion

This study investigated the first RHD outbreak in Kwara State, Nigeria. The disease has spread to other parts of Nigeria. Hence, a nationwide active surveillance of RHD should be conducted. Nigeria must immediately evaluate the efficacy and immunogenicity of the available RHD vaccines and advise farmers accordingly to prevent the huge economic impact of TADs such as RHD.

## Data Availability Statement

The raw data supporting the conclusions of this article will be made available by the authors without undue reservation.

## Ethics Statement

Ethical clearance was obtained from the ethical review board of the Kwara State Ministry of Agriculture and Rural Development (MoARD), Ilorin-Nigeria (reference number: VKW/ERB/OM/02/77). Informed consent was sought from the respondents and participants could opt-out at any time.

## Author Contributions

AA-M and LA were involved in planning the study and data collection. AA-M and MO drafted the initial manuscript. All authors did the overall review of the manuscript, read, and approved the final manuscript.

## Conflict of Interest

The authors declare that the research was conducted in the absence of any commercial or financial relationships that could be construed as a potential conflict of interest.

## Publisher's Note

All claims expressed in this article are solely those of the authors and do not necessarily represent those of their affiliated organizations, or those of the publisher, the editors and the reviewers. Any product that may be evaluated in this article, or claim that may be made by its manufacturer, is not guaranteed or endorsed by the publisher.

## References

[1] Liu SJ Xue HP Pu BQ Qian NH. A new viral disease in rabbit. Anim Husb Vet Med. (1984) 16:253–5.

[2] Wy X. Viral haemorrhagic disease of rabbits in the People's Republic of China: epidemiology and virus characterization. Rev Sci Tech. (1991) 10:393–408. 10.20506/rst.10.2.559 1760583

[3] World Organization for Animal Health (OIE). Rabbit Haemorrhagic Disease, Benin. (2015). Available online at: https://www.oie.int/wahis_2/public/wahid.php/Reviewreport/Review?page_refer=MapEventSummary&reportid=19903 (accessed December 15, 2020).

[4] Erfan AM Shalaby AG. Genotyping of rabbit hemorrhagic disease virus detected in diseased rabbits in Egyptian Provinces by VP60 sequencing. Vet World. (2020) 13:1098–107. 10.14202/vetworld.2020.1098-1107 32801560 PMC7396351

[5] Ismail MM Mohamed MHA El-Sabagh IM Al-Hammadi MA. Emergence of new virulent rabbit hemorrhagic disease virus strains in Saudi Arabia. Trop Anim Health Prod. (2017) 49:295–336. 10.1007/s11250-016-1192-5 27913973

[6] Elliott S Saunders R. Rabbit haemorrhagic disease in the UK. Vet Rec. (2017) 181:516–6. 10.1136/vr.j5149 29127178

[7] Abrantes J Lopes AM Dalton KP Melo P Correia AA Ramada M. New variant of rabbit hemorrhagic disease virus, Portugal, 2012-2013. Emerg Infect Dis. (2013) 19:1900–2. 10.3201/eid1911.130908 24206671 PMC3837648

[8] Asgari S Hardy JR Sinclair RG Cooke BD. Field evidence formechanical transmission of rabbit haemorrhagic disease virus (RHDV) by flies(*DipteraCalliphoridae*) among wild rabbits in Australia. Virus Res. (1998) 54:123–32. 10.1016/S0168-1702(98)00017-3 9696121

[9] World Organisation for Animal Health. Chapter 13.2. Rabbit haemorrhagic disease. In:Terrestrial Animal Health Code, 27th ed. Paris: OIE (2018).

[10] Gleeson M Petritz O. Emerging infectious diseases of rabbits. Vet Clincis North Am Exot Anim Pract. (2020) 23:249–61. 10.1016/j.cvex.2020.01.008 32327034

[11] Ohio Department of Agriculture (2018). Available online at: https://www.agri.ohio.gov via https://www.medvetforpets.com/rabbit-hemorrhagic-disease-faq/ (accessed August 16, 2021).

[12] International Committe on Taxonomy of Viruses. Calciviridae (2020). Available online at: www.ictv.com (accessed December 15, 2020).

[13] Rocchi MS Dagleish M. Diagnosis and prevention of rabbit viral haemorrhagic disease 2. In Pract. (2018) 40:11–6. 10.1136/inp.k5428850992

[14] Abrantes J van der Loo W Le Pendu J Esteves PJ. Rabbit haemorrhagicdisease (RHD) and rabbit haemorrhagic disease virus (RHDV): a review. Vet Res. (2012) 43:12. 10.1186/1297-9716-43-12 22325049 PMC3331820

[15] World Organization for Animal Health (OIE). Rabbit Hemorrhagic Disease. (2020). Available online at: https://www.aphis.usda.gov/publications/animal_health/fs-rhdv2.pdf (accessed December 15, 2020).

[16] World Organization for Animal Health (OIE). Rabbit Hemorrhagic Disease, Nigeria. (2020). Available online at: https://www.oie.int/wahis_2/public/wahid.php/Reviewreport/Review?page_refer=MapFullEventReport&reportid=36114&newlang=en (accessed December 15, 2020).

[17] RHD virus hits Nigeria, government government urged on actions. PM News. (2020). Available online at: https://www.pmnewsnigeria.com/2020/08/17/rhd-virus-hits-nigeria-government-urged-on-actions/ (accessed December 15, 2020).

[18] Ambagala A Ababio P Lamboo L Goolia M Lung O Berhane Y. Outbreak of rabbit hemorrhagic disease virus 2 infections, Ghana. Emerg Infect Dis. (2021) 27:1999. 10.3201/eid2707.210005 34153219 PMC8237895

[19] Happi AN Ogunsanya OA Oguzie JU Oluniyi PE Olono AS Heeney JL. Microbial metagenomic approach uncovers the first rabbit haemorrhagic disease virus genome in Sub-Saharan Africa. Scientific reports. (2021) 11:1–8. 10.1038/s41598-021-91961-2 34210997 PMC8249450

[20] Chah J Attamah C Nnodim M. Disease management practices among rabbit farmers in Enugu State Nigeria. J Agric Extension. (2018) 22:130. 10.4314/jae.v22i3.13

[21] World Health Organization. Antimicrobial Resistance. (2020). Available online at: https://www.who.int/news-room/fact-sheets/detail/antimicrobial-resistance (accessed December 15, 2020).

[22] Baratelli M Molist-Badiola J Puigredon-Fontanet A Pascual M Boix O Mora-Igual F. Characterization of the maternally derived antibody immunity against Rhdv-2 after administration in breeding does of an inactivated vaccine. Vaccines. (2020) 8:484. 10.3390/vaccines8030484 32872139 PMC7564433

[23] Müller C Ulrich R Schinköthe J Müller M Köllner B. Characterization of protective humoral and cellular immune responses against RHDV2 induced by a new vaccine based on recombinant baculovirus. Vaccine. (2019) 37:4195–203. 10.1016/j.vaccine.2019.04.061 31182325

[24] Kerr PJ Donnelly T. Viral infections of rabbits. Vet Clincis North Am Exot Anim Pract. (2013) 16:437–68. 10.1016/j.cvex.2013.02.002 23642871 PMC7110462

[25] Martin-Alonso A Martin-Carrillo N Garcia-Livia K Valladares B Foronda P. Emerging rabbit haemorrhagic disease virus 2 (RHDV2) at the gates of the African continent. Infect Genet Evol. (2016) 44:46–50. 10.1016/j.meegid.2016.06.034 27321441

